# Supplementary material for: Dynamics of specialization in neural modules under resource constraints
Source: Nat Commun. 2025 Jan 2;16:187. doi: 10.1038/s41467-024-55188-9 (PMC11695987; doi:10.1038/s41467-024-55188-9)
Supplement: Supplementary file 1 — Supplementary Information [file 41467_2024_55188_MOESM1_ESM.pdf]

DYNAMICS OF SPECIALIZATION IN NEURAL MODULES UNDER RESOURCE CONSTRAINTS

Gabriel Béna*
g.bena21@imperial.ac.uk

Dan F. M. Goodman*
d.goodman@imperial.ac.uk

November 7, 2024

1 Supplementary Materials

1.1 Q measure derivation

[1] defines the modularity measure Q for an undirected graph. We modify the definition for a directed graph as:

$$Q = \frac{1}{M}\sum_{ij}(A_{ij} - P_{ij})\delta_{g_i g_j} = \frac{1}{M}\sum_{ij}\left(A_{ij} - \frac{k_i^{out}k_j^{in}}{M}\right)\delta_{g_i g_j} \tag{1}$$

where M is the total number of edges in the network, A is the adjacency matrix, δ is the Kronecker delta, g_i is the group index of node i, k_i^{out}/k_j^{in} are the out-degree/in-degree of node i/j respectively, used to compute P_{ij}: the probability of a connection between nodes i and j if we randomized the edges respecting node degrees. For our network, we have 2 sub-networks (with group index 0 and 1 respectively) of n neurons, densely connected, with a fraction p of active inter-connections between the two. From this we get:

$$M = 2n^2(1+p)$$

$$\forall i \in [1, 2n], \quad g_i = \begin{cases} 0, & \text{if } i \in [0, n-1] \\ 1, & \text{if } i \in [n, 2n] \end{cases}$$

$$\forall (i,j) \in [0, 2n]^2, \quad A_{i,j} = \begin{cases} 1, & \text{if } \delta_{g_i g_j} = 1 \\ 0 \text{ or } 1, & \text{if } \delta_{g_i g_j} = 0 \end{cases}$$

$$\forall (i,j) \in [0, 2N]^2, \quad \begin{cases} k_i^{out} = \sum_{j'} A_{ij'} \approx n(1+p) \\ k_j^{in} = \sum_{i'} A_{i'j} \approx n(1+p) \end{cases}$$

$$\text{And} \quad \begin{cases} \sum_i k_i^{out} = \sum_i \sum_{j'} A_{ij'} = n^2(1+p) \\ \sum_j k_j^{in} = \sum_j \sum_{i'} A_{i'j} = n^2(1+p) \end{cases}$$

$$\text{Thus: } Q = \frac{1}{2n^2(1+p)}\sum_{ij}\left(A_{ij} - \frac{(1+p)}{2}\right)\delta_{g_i g_j}$$

$$Q = \frac{1}{2n^2(1+p)}(2n^2)\left(1 - \frac{(1+p)}{2}\right)$$

$$\boxed{Q = \frac{1-p}{2(1+p)}}$$

*Imperial College London

1.2 Network Accuracy

Supplementary Figure 1 displays the network's accuracy for results 3.1. We always test on a separate testing set, and train for a fixed number of epochs to avoid overfitting, repeated for 10 random seeds. We can see that, when the sparsity of communications is very high, networks only manage to perform slightly above chance (∼60%). This shows that the task is not trivial for networks of that size, which was one of our objectives so as to not over-parameterize the networks and make the results meaningful.

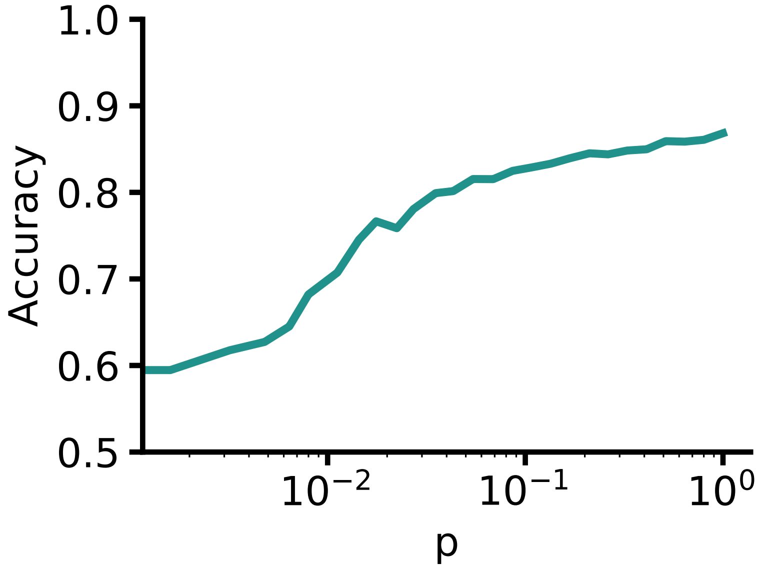

Supplementary Figure 1: Accuracy for networks composed of 25-neurons modules, with varying levels of inter-module communication p. Results are shown for 5 independent experiments per parameter-set (sparsity), with confidence intervals showed as shaded areas.

1.3 Decision Dynamics

Supplementary Figure 2 showcases decision dynamics over the course of training, for more or less modular networks, demonstrating the point made in section 3.2. We see that the more interconnected, the more the networks tend to rely on a single decision-taking agent (decision going up to 1 or falling closer to 0 on average) Targets correspond to the two targets of equation (2), and we can see that the small bias discussed when presenting the tasks is present at every sparsity, but getting ever more present in more interconnected networks.

1.4 Parameter sweeps for structure-function relationship

Figure 3 shows the complete parameter sweeps referred to in section 3.2 of the main text.

1.5 Code examples

1.5.1 Noisy inputs

In section 3.3, we investigate the different dynamics of functional specialization in networks unrolled in time. To that end, we add noisy dynamics to the input, which are by default identical at every time-steps, and create a noisy version of the dataset. For each training batch, and at each time-step, we add an average from a few other random samples of the same batch, multiplied by a noise-level. Example code is provided here This would have to be repeated at each time-step (see following section).

```
def add_structured_noise(batch_t, n_samples=5, noise_ratio=0.9):
    noised_idxs = np.stack(
        [
            np.random.choice(batch_t.shape[0], size=n_samples, replace=False)
            for _ in range(batch_t.shape[0])
```

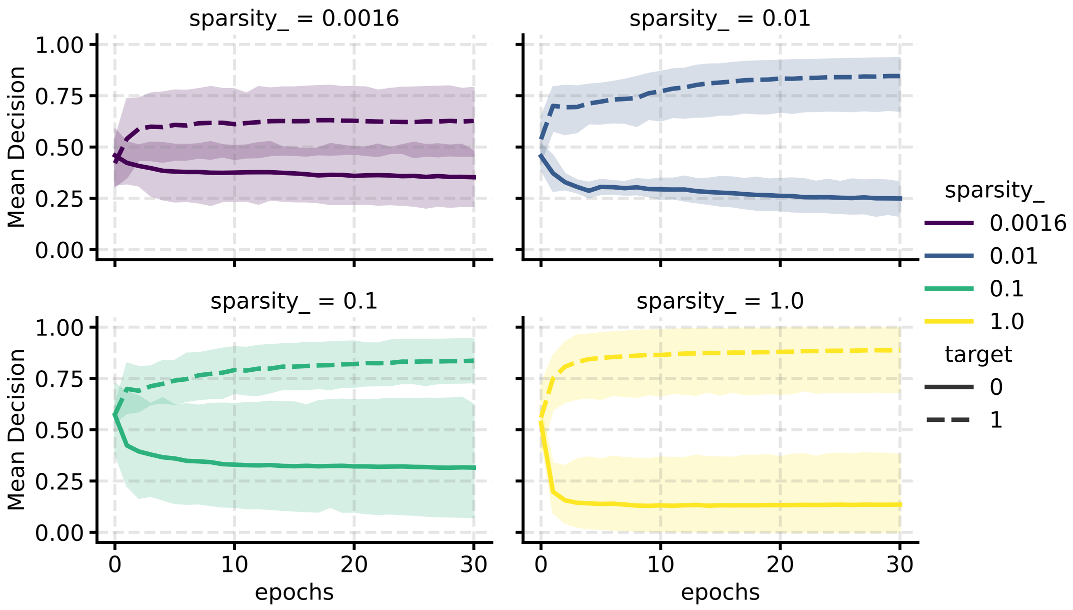

Supplementary Figure 2: Decision-making dynamics over training, for networks composed of 25 neurons per module. Lines represent the average decision making in networks (1 being always module 1 and 0 always module 0). Results are shown for 5 independent experiments per parameter-set (sparsity), with confidence intervals showed as shaded areas.

```
        ]
    )
    noisy_samples = batch_t[noised_idxs] * (
        np.random.rand([n_samples] + list(batch_t.shape[1:])) < (1 / n_samples)
    )
    noisy_batch = (1 - noise_ratio) * batch_t + noise_ratio * noisy_samples.mean(1)
    return noisy_batch
```

1.5.2 Dynamic inputs

Here is how we create input with noisy dynamics, and potentially stochastic start times.

```
def create_stochastic_batch(batch, n_samples, noise_ratio, random_start) :
    noisy_batch = np.stack(
        [add_structured_noise(batch_t, n_samples, noise_ratio) for batch_t in batch]
    )
    if random_start :
        start_times = np.random.randint(1, batch.shape[0] - 1, (batch.shape[1],))
        mask = (
                    np.arange(nb_steps)[:, None]
                    >= start_times[None, :,]
                )
         pure_noise = np.stack(
            [add_structured_noise(batch_t, n_samples, 1.0) for batch_t in batch]
        )

        stochastic_timing_batch = noisy_batch * mask + pure_noise * (not mask)
        return stochastic_timing_batch
    else :
        return noisy_batch
```

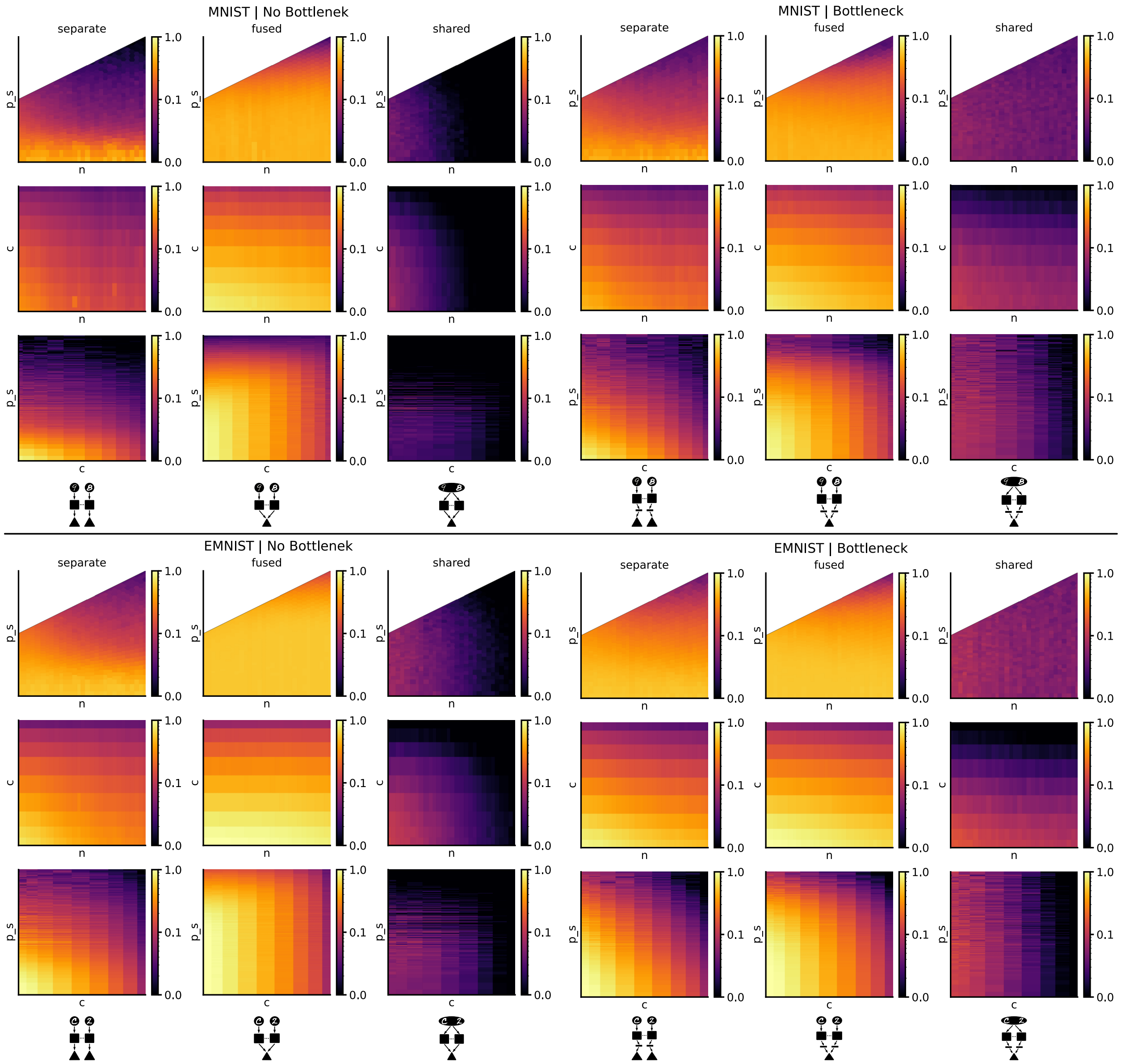

Supplementary Figure 3: Specialization for all architectures and data. See caption to figure 3.

References

[1] Newman, M. E. J. Modularity and community structure in networks. *Proceedings of the National Academy of Sciences* **103**, 8577–8582 (2006). URL `https://www.pnas.org/content/103/23/8577`. Publisher: National Academy of Sciences Section: Physical Sciences.
